# The Analysis of Three-Body Contact Temperature under the Different Third Particle Size, Density, and Value of Friction

**DOI:** 10.3390/mi8100302

**Published:** 2017-10-11

**Authors:** Horng-Wen Wu, Yang-Yuan Chen, Jeng-Haur Horng

**Affiliations:** 1 Department of Systems and Naval Mechatronic Engineering, National Cheng Kung University, Tainan 701, Taiwan; z7708033@email.ncku.edu.tw (H.-W.W.); td7211@gmail.com (Y.-Y.C.); 2 Department of Power Mechanical Engineering, National Formosa University, Yunlin 632, Taiwan

**Keywords:** friction, wear, surface roughness, three-body micro-contact, contact temperature

## Abstract

Recently, many studies have investigated the friction, wear, and temperature characteristics of the interface between two relative movements. Such analyses often set the coefficient of friction as a fixed value and are analyzed in cases of two-body contact; however, the interface is often a three-body contact and the coefficient of friction varies depending on the operating conditions. This is a significant error in the analysis of contact characteristics, therefore, in this study, the actual interface and the change of the coefficient of friction were analyzed based on three-body micro-contact theory where the contact temperature was also analyzed and the difference between the generally assumed values were compared. The results showed that under three-body contact, the coefficient of total friction increased with an increase in particle size; and at a different particle size and area density of particles, the surface contact temperature increased with the plasticity index and load increases, and the particle contact temperature increased with the increasing particle size. The surface temperature rise was mainly affected by the ratio of the average temperature between surface 1 and surface 2 to the multiplication between the 100th root of the area density of particles and the square root of the equivalent surface roughness (*T_s_*_1*s*2_ave_^*^/*η_a_*^0.01^*σ*^0.5^) and the ratio of the 10th root of the mean particle diameter to the 100th root of the equivalent surface roughness (*x_a_*^0.1^/*σ*^0.001^). Particle temperature was mainly affected by the ratio of the 10th root of the mean particle diameter to the 100th root of the equivalent surface roughness (*x_a_*^0.1^/*σ*^0.001^) and the area density of particles *η_a_*. Our study indicated that when the contact of surface with surface and the contact of the particles with the surface, the resulting heat balance was assigned to the particles and the surface in a three-body contact situation. Under this contact behavior, it could avoid a too high a rise in micro-contact temperature to achieve the material failure temperature.

## 1. Introduction

When the third bodies (particles) are present between the contact interfaces of two surfaces, there are two kinds of contact points, including surface-to-surface contact spots and particle-to-surface contact spots, in the roughness of the surfaces. The contact temperature at the interface is an important influencing factor in the performance of motion components where the main heat source is friction heat. As the surface is rough, the contact area that accepts the heat is very small; therefore, the micro-contact temperature (bulk temperature + flash temperature) is the main factor affecting machine characteristics. Many contact temperature analyses have been proposed. Blok [1] suggested that moving the heat source would cause the contact temperature to rise in the interface. In 1942, Jaeger [2] described that predicting the occurrence of scuffing could be calculated by the flash temperature formula. Tian et al. [3] considered the heat partition between two contacting bodies, and solutions of the interface flash temperature were presented for a general sliding contact case, as well as for the sliding contact between two moving asperities. Knothe and Liebelt [4] demonstrated that the different surface roughness could be caused the maximum contact temperature rise. Bansal and Streator obtained the complete temperature and heat partition distributions in the interface by a linear regression method [5], and in 2012, proposed to predict the maximum temperature rise formulae for larger elliptical ratios [6]. In 2015, Kennedy et al. [7] discussed how the analysis of the contact temperature of pin-on-disk contacts between thermoplastic polymer pins and metallic or ceramic disks could determine the maximum allowable sliding velocities during tribotesting. However, few studies have analyzed the contact temperature when the particle presents an interface between two surfaces.

The real contact area is an important factor affecting the contact temperature rise in the interface, and this has been studied extensively between two rough surfaces, including the Greenwood and Williamson proposed GW model [8], the Pullen and Williamson proposed PW model [9], the Chang et al. proposed CEB model [10], the Horng proposed H model [11], the Zhao et al. proposed ZMC model [12], and the Kogut and Etsion proposed KE model [13]. However, the third particle is often in contact with the interface of two surfaces during component operation. Bogy et al. [14,15,16] and Stachowiak [17] discussed the impacts of the particles on wear, contact force, and contact temperature rise between the hard disk contact surfaces. Horng [18] described the contact characteristics of rough surfaces when the contact interfaces show three-body contact behavior. Sundh and Olofsson [19] indicated the transient behavior of the contact conditions in terms of wear transitions and elevated contact temperatures. In 2016, Pu et al. [20] proposed that a friction and flash temperature prediction approach could be employed as an engineering tool for the performance improvement of spiral bevel and hypoid gears, and other transmission components with the same characteristics. In 2016, Narayanaswamy et al. [21] experimentally showed the specific wear rate of the pearlitic microstructure decreased with a reduction in the abrasive particle size, irrespective of the particle type. In 2017, Costa et al. [22] experimentally showed the addition of alumina particles contributed to a significant reduction in the friction coefficient, in particular for the largest particles. Kennedy et al. [23] indicated that oxidation had occurred within the sliding contacts as a result of high sliding contact temperatures, which also enabled oxide debris and worn material to be transferred from the counterface and incorporated into a mechanically-mixed tribolayer on the surface. The hard tribolayer contributed to the reduction in wear rate at high sliding velocities. Therefore, this work combined the contact model, friction model, and contact temperature model to analyze the contact temperature rise characteristics in three-body contact situations at various particle sizes, area density of particles, relative speeds, surface roughness values, and applied loads.

## 2. Theoretical Analysis

### 2.1. Micro-Contact Model

**Figure 1:**
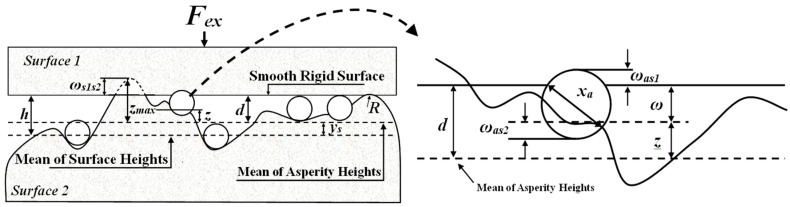
Geometry of three contacting bodies.

The two rough asperities can be transformed into the contact of a plane with an equivalent rough surface [10], and Figure 1 shows the geometry of the three contacting bodies [24]. In the contact model, the following assumptions are made:

1. The contact interface is under dry friction conditions.
2. The peak of the surface asperity is hemispherical and the same radius of curvature (*R*) and the Gaussian distribution *φ*(*z*) shows the change of the asperity height.
3. All surface asperities are separate by a far distance and there is no interaction between them.
4. There is no bulk deformation, but the surface asperities deform during contact.
5. The shape of the third particle is spherical, the mean diameter is *x_a_*, and the Gaussian distribution *φ_a_*(*x*) shows the change of the particle diameter.
6. The particles are much harder than surface 1 and surface 2 due to work hardening, and both the surfaces deform plastically during contact with particles. This means that the size of the particles remains constant during the operation.
7. The slopes of surface asperities are negligibly small.

The distribution of the particle diameters *φ*_a_(*x*) and asperity heights *φ*(*z*) of rough surfaces are assumed to be a Gaussian distribution and are given as:

(1) $$\varphi_{a}(x)=\frac{1}{\sqrt{2\pi }\times \sigma_{a}}\exp\left[-0.5{\left(\frac{x-x_{a}}{\sigma_{a}}\right)}^{2}\right]$$

(2) $$\varphi (z)=\frac{1}{\sqrt{2\pi }\times \sigma_{s}}\exp\left[-0.5{\left(\frac{z-\sigma }{\sigma_{s}}\right)}^{2}\right]$$

where *σ_a_* is the standard deviation of the particle diameter, *σ_s_* is the standard deviation of the asperity heights, and *σ* is the equivalent RMS of the contacting surface roughness.

Wear debris or foreign particles are often present between two surfaces so that the contact interface forms a three-body contact system in the relative movement of the components during operation. The total contact load (*F_total_*) is borne by the particle-to-surface (*F_s_*_1*a*_) and surface-to-surface (*F_s_*_1*s*2*-s*1*a*_) contact spots. *A_total_* includes *A_s_*_1*s*2*-s*1*a*_ and *A_s_*_1*a*_, where *A_s_*_1*s*2*-s*1*a*_ is the real contact area for surface-to-surface contact and *A_s_*_1*a*_ is the real contact area for particle-to-surface contact. Based on the H model [18], the total contact load *F_total_* and the total contact area *A_total_* are given by the following equations:

(3) $$\begin{array}{ll} F_{total} & =F_{s1a}+F_{s1s2-s1a}=\frac{\pi H_{s1}H_{s2}\eta_{a}A_{n}}{H_{s1}+H_{s2}}\left[\frac{9\pi^{2}}{4}\left(\frac{H_{s1}^{2}}{E_{as1}^{2}}+\frac{H_{s2}^{2}}{E_{s1s2}^{2}}\right)\int_{d-h_{e}}^{d}{x_{a}}^{2}\varphi_{a}(x)dx+\int_{d}^{x_{\max}}{x_{a}}^{2}\varphi_{a}(x)dx\right]\\ & +\left(1-\frac{\pi H_{s1}\eta_{a}}{H_{s1}+H_{s2}}\int_{X_{\min}}^{X_{\max}}{x_{a}}^{2}\varphi_{a}(x)dx\right)\cdot F_{s1s2} \end{array}$$

(4) $$A_{total}=A_{s1a}+A_{s1s2-s1a}=\frac{\pi H_{s2}\eta_{a}A_{n}}{H_{s2}+H_{s1}}\left[\frac{9\pi^{2}}{4}\left(\frac{H_{s1}^{2}}{E_{as1}^{2}}+\frac{H_{s2}^{2}}{E_{s1s2}^{2}}\right)\int_{d-h_{e}}^{d}{x_{a}}^{2}\varphi_{a}\left(x\right)dx+\int_{d}^{X\max}{x_{a}}^{2}\varphi_{a}\left(x\right)dx\right]\;+A_{s1s2}\cdot \left\{1-\frac{\pi H_{s1}\eta_{a}}{H_{s1}+H_{s2}}\int_{d}^{X_{\max}}{x_{a}}^{2}\varphi_{a}(x)dx\right\}$$

where *A_n_* is the nominal contact area; *H_s_*_1_ is the hardness of surface 1; *H_s_*_2_ is the hardness of surface 2; *E_s_*_1*s*2_ is the equivalent elastic modulus of surfaces 1 and 2; *E_s_*_1*a*_ is the equivalent elastic modulus of surfaces 1 and the particle; *d* is the separation based on asperity heights; *η_a_* is the area density of particles; *X*_max_ is the maximum particle size; and *h_e_* is the maximum separation of two surfaces with particles that leads to plastic contact. When the particle diameter *x_a_* = 0, Equations (3) and (4) become *F_total_* = *F_s_*_1*s*2_ and *A_total_* = *A_s_*_1*s*2_, as per the ZMC two-body micro-contact model [12].

**Figure 2:**
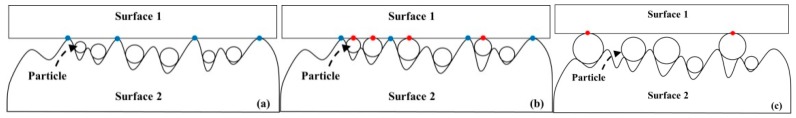
The contact behavior of wear debris between the contact interface (**a**) surface-to-surface two-body contact; (**b**) three-body contact; and (**c**) particle-to-surface two-body contact.

There are three contact behaviors when there are particles present in the interface, as shown in Figure 2. Figure 2a shows the surface-to-surface two-body contact, which occurs when the particle diameter is smaller or there are fewer particles per unit area where the particle sinks into the trough and the load is completely borne by the peak. Figure 2b shows three-body contact, which occurs when the number of particles per unit area and load are larger, so the load is borne by the particles and the peak, respectively. Figure 2c displays particle-to-surface two-body contact, which is when the particle diameter and the number of particles per unit area are larger, so the surface is distracted by the particles and the load is completely borne by the particles. The Gaussian distribution *φ_a_*(*x*) shows the change of the particle diameter, and the number of real contact particles *n_a_* can be expressed as follows [25]:

(5) $$n_{a}=\eta_{a}\times A_{n}\int_{d}^{X_{\max}}\varphi_{a}(x)dx$$

The average particle contact load *F_ai_* can be obtained by Equations (3) and (5):

*F_ai_* = *F_s1a_*/*n_a_* (6)

### 2.2. Friction Model

The process and mechanism of friction are important and complex, and many previous studies have put forward theories for causes of friction. According to Bhushan and Nosonovsky [26,27], the friction is expressed as the sum of five components: surface summit deformation (*μ_s_*), plowing deformation friction by particles entrapped between contact surfaces (*μ_p_*), particle adhesion friction (*μ_pa_*), adhesion friction (*μ_a_*), and ratchet friction (*μ_r_*) at the contact region. The total coefficient of friction *μ_total_* and friction components become:

(7) $$\mu_{total}=(\mu_{s}++\mu_{a}+\mu_{r})+(\mu_{p}+\mu_{pa})=\mu_{s1s2}+\mu_{s1a}\;=\frac{\left[A_{s1s2-s1a}\tau_{a}+A_{s1s2-s1a}\tau_{s1s2}+A_{s1s2-s1a}\tau_{a}\times {\left(\sigma /\beta \right)}^{2}]+(A_{s1a}\tau_{s1a}+A_{s1a}\tau_{a}\right)}{F_{total}}$$

where *μ_s_*_1_*_s_*_2_ is coefficient of friction of surfaces 1 and 2; *μ_s_*_1*a*_ is the coefficient of friction of surface 1 and the particle; *τ*_a_, *τ_s_*_1*s*2_, and *τ_s_*_1*a*_ are the shear stress of adhesion, the shear stress of deformation between surfaces, and the shear stress of deformation between the particles and the surface, respectively. *τ_a_* is in accordance with [28] using the shear stress of adhesion *τ_a_* = *G*/1000, and *τ_s_*_1*s*2_ and *τ_s_*_1*a*_ are in accordance with [27] using $H=3\sqrt{3}\tau$ *A_s_*_1*s*2*-s*1*a*_ and *A_s_*_1*a*_ are the real areas of contact during two-surface deformation and particle-surface 1 deformation, respectively, and are calculated from Equation (4).

### 2.3. Flash Temperature Model

Friction occurs when surfaces 1 and 2 contact with particles at the relative speed *V*. The following assumptions are given for simplifying the heat transformation problems.

1. The heat generated by friction is considered to be a moving heat source at a steady condition.
2. The heat source is uniform and circular.
3. Between the contact interfaces, the particles themselves do not rotate or move.

On the basis of the above assumptions, the energy generated by friction between particles and the surface energy is converted into heat. The heat conductance of a unit area is used to express the magnitude of the heat conductance:

(8) $$q=\frac{Q}{A}=\frac{\mu_{total}F_{total}V}{\pi a^{2}}=\mu_{total}PV$$

where *Q* is the amount of heat generated at the contact spots; *P* is the average contact pressure; *V* is the relative speed; *F_total_* is the normal load; *A* is the real single contact area; and *a* is the contact radius. The Péclet number (*P_e_*) is a non-dimensional speed parameter that is used to evaluate the movement rate of contact heat and is defined as follows:

(9) $$P_{e}=\frac{Va\rho C_{p}}{2K}$$

where *K* is the heat conductance coefficient; *ρ* is the density; and *C_p_* is the specific heat. Different Péclet numbers exist at different velocities. Tian and Kennedy [3] proposed a model with a maximum temperature that can be applied to all Péclet numbers. In this model, the average temperature increase of the spherical contact heat is expressed as follows:

(10) $$T=\frac{1.22qa}{K\sqrt{\pi (0.6575+P_{e})}}$$

If *q* is the heat flux between two surfaces in contact, *R_w_ q* is the heat of the incoming surface 1 and (1 – *R_w_*) *q* is the heat of the incoming surface 2. *R_w_* is the thermal distribution factor, given by the following equation:

(11) $$R_{w}=\frac{1}{1+\frac{K_{s1}}{K_{s2}}\sqrt{\frac{0.6575+P_{e,s1}}{0.6575+P_{e,s2}}}}$$

where *K_s_*_1_ is the thermal conductivity of surface 1; *K_s_*_2_ is the thermal conductivity of surface 2; *P_e,s_*_1_ is the Péclet number of surface 1; and *P_e,s_*_2_ is the Péclet number of surface 2. Substituting Equation (11) into Equation (10) yields the following equation:

(12) $$T=\frac{1.22qa}{\sqrt{\pi }\left[K_{s2}\sqrt{\left(0.6575+P_{e,s2}\right)}+K_{s1}\sqrt{\left(0.6575+P_{e,s1}\right)}\right]}$$

The temperature rise (*T*) is a flash temperature generated at a single contact spot. The equation for the flash temperature at a single contact spot is as follows:

(13) $$T_{s1s2}=\frac{1.22q_{s1s2}a_{s1s2}}{\sqrt{\pi }\left[K_{s2}\sqrt{0.6575+P_{e,s2}}+K_{s1}\sqrt{0.6575+P_{e,s1}}\right]}$$

Consequently, the average contact temperature of asperities between surface 1 and surface 2 is expressed as follows:

(14) $$T_{s1s2,ave}=\frac{\int_{d}^{Z_{\max}}T_{s1s2}\varphi (z)dz}{\int_{d}^{Z_{\max}}\varphi (z)dz}$$

where *z*_max_ is the maximum asperity height. The flash temperature between a single particle and surface 1 is expressed as follows [29]:

(15) $$T_{s1a}=\frac{1.22\mu_{s1a}V\sqrt{F_{ai}H_{s2}}}{\pi \left[K_{s2}\sqrt{\left(0.6575+P_{e,s2}\right)}+K_{a}\sqrt{\left(0.6575+P_{e,a}\right)}\right]}$$

where *F_ai_* is the average contact load of particle (*F_ai_* = *F_s_*_1*a*_/*n_a_*); and *P_e,a_* is the Péclet number of the particle. Therefore, the average contact temperature between the particle and surface is expressed as follows:

(16) $$T_{s1a,ave}=\frac{\int_{x_{\min}}^{x_{\max}}T_{s1a}\varphi_{a}(x)dx}{\int_{x_{\min}}^{x_{\max}}\varphi_{a}(x)dx}$$

## 3. Results and Discussion

In the past, many analyses of heat flow with contact surfaces assumed that the coefficient of friction had to be fixed. For example, Bulsara [25] analyzed the average contact temperature of abrasive particles and Rolland [30] analyzed the case of the Swiss lever escapement mechanism. However, the coefficient of friction of the actual contact surface changes with the changing of the parameters, so the results of the analysis and the actual situation may contain errors. In this paper, the coefficient of friction is assumed to be fixed to 0.1 and the coefficient of friction changes with the contact conditions were analyzed, indicating the differences in the contact temperature. SUJ2 and CrMo steel were used for surfaces 1 and 2, and the particles in this analysis, and the properties are listed in Table 1. Under the three-body contact situation, there are two kinds of contact temperature: surface peak with surface peak (*T_s_*_1*s*2*,ave*_), and particle with surface peak (*T_s_*_1*a,ave*_). The temperature characteristics are expressed as described in [29]:

(17) $$T_{s1s2,ave}^{*}=\frac{T_{s1s2,ave}\times K_{s2}}{H_{s2}\times \alpha_{s2}}$$

(18) $$T_{s1a,ave}^{*}=\frac{T_{s1a,ave}\times K_{s2}}{H_{s2}\times \alpha_{s2}}$$

where *K_s_*_2_, *H_s_*_2_, and *α_s_*_2_ are the thermal conductivity of surface 2, the hardness of surface 2, and the thermal diffusivity of surface 2, respectively. *α_s_*_2_ = *K_s_*_2_/(*ρ_s_*_2_ × *C_ps_*_2_), where *ρ_s_*_2_ and *C_ps_*_2_ are the density of surface 2 and specific heat capacity of surface 2, respectively. The material pair used in the analysis was SUJ2 and CrMo, which are types of steel commonly used for bearings, gears, and ball screws.

**Figure 3:**
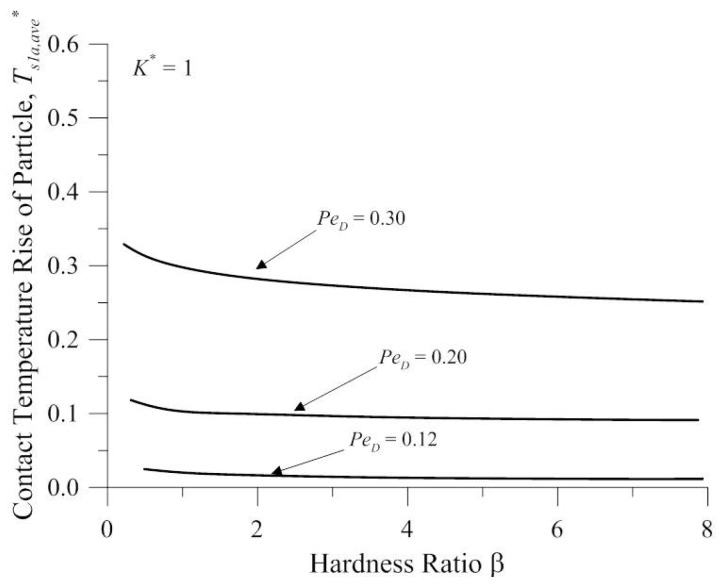
The contact temperature vs. the hardness ratio *β* at various Peclet numbers based on the particle diameter *Pe_D_* for the results from the present analysis.

Figure 3 shows the contact temperature vs. the hardness ratio β at various Peclet numbers based on the particle diameter *Pe_D_* for the results from the present analysis. Where K^*^ = *K_s_*_1_/*K_a_*, the Peclet number is based on the particle diameter *Pe_D_* = *Vx_a_*/*α_s_*_1_, where *α_s_*_1_ is the thermal diffusivity of surface 1, and the hardness ratio β = *F_ai_*/(0.5 × π × *r_a_*^2^ × *H_s_*_2_), where *r_a_* is the contact radius of surface 1 and the particle. This Figure shows that the contact temperature rise of the particle increases with the increasing Peclet number based on the particle diameter *Pe_D_*, which decreases as the hardness ratio increases. The trend of the variation of *T_f_* is similar with the results of Khonsari et al. [29]. However, the reference was to predict the scuffing phenomenon, so the operating conditions are relatively more severe than the conditions of present analysis.

**Figure 4:**
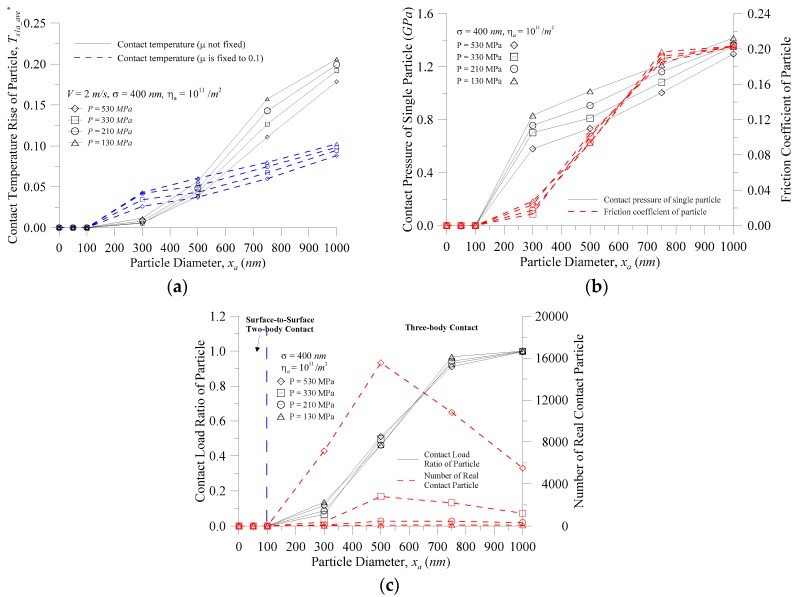
The characteristics of the particle vs. the particle diameter at various contact pressures for (**a**) the contact temperature rise; (**b**) the contact pressure of a single particle and the coefficient of friction; and (**c**) the contact load ratio and the real contact number.

Figure 4 shows the characteristics of the particle vs. the particle diameter at various contact pressures for (a) the contact temperature rise; (b) the contact pressure of a single particle and the coefficient of friction; and (c) the contact load ratio of the particle (*F_s_*_1a_^*^ = *F_s_*_1*a*_/*F_total_*) and the number of real contact particles when the equivalent RMS of the contacting surface roughness *σ* = 400 nm, *η_a_* = 10^11^/m^2^, and *V* = 2.0 m/s. As shown in Figure 4a, however, whether the coefficient of friction was assumed to be fixed (*μ* = 0.1) or variable, the contact temperature rise of the particles increased as the particle size increased. However, when the particle size was greater than 500 nm, the actual contact temperature was higher than the contact temperature when the coefficient of friction was assumed to be fixed, and the difference was larger as the particle size increased; at a particle size of 750 nm, the difference reached 85%–96%. The reason for this is that the contact temperature is varied and so can be illustrated in Figure 4b where the friction heat flux is *μPV*. At the same speed, the coefficient of friction of the particle and the contact pressure of a single particle (Figure 4b) increased with the increasing particle size, and the main reason for the rapid increase in the contact temperature is the coefficient of friction of the particles. Figure 4a also showed that with the same particle size, the greater the contact pressure, the smaller the contact temperature rise of the particle. The reason for this is that, at the smaller contact pressure, the particles easily distracted the two surfaces so that the external load was mainly borne by the minority particles, as shown in Figure 4c. At the same time, as that shown in Figure 4b, the contact pressure of a single particle increased with decreases in the contact pressure at the same particle size. Furthermore, at *x_a_* < 100 nm, the contact pressure of a single particle was reduced to 0, and is explained in Figure 4c, which shows that the contact load ratio of particles and number of contact particles were zero at *x_a_* < 100 nm. As the space of the interface was much larger than the particle diameter, the particles sank into the trough, and there was no particle contact between the contact interfaces. As illustrated in Figure 2, when the particle appeared in the interface, there are three kinds of contact behavior that can correspond to the contact load ratio of particles (*F_s_*_1a_^*^): (1) as *F_s_*_1a_^*^ = 0, the surface-to-surface two-body contact appears; (2) as *F_s_*_1a_^*^ = 1, the contact behavior is a particle-to-surface two-body contact; and (3) for 0 < *F_s_*_1a_^*^ < 1, three-body contact exists. Under this operating condition, the black solid line in Figure 4c displays that when *x_a_* < 100 nm, the surface-to-surface two-body contact appeared; as the particle size increased, the contact behavior entered the three-body contact until the particle size reaches 1000 nm; and when the contact load ratio of a particle reached 100%, the contact behavior entered the particle-to-surface two-body contact zone. The red dotted line in Figure 4c shows that the number of real contact particles increased as the contact pressure increased. With the same contact pressure, the number of real contact particles increased with an increase in the particle size, and the contact behavior entered the three-body contact zone at a particle size greater than 100 nm. However, when *x_a_* > 500 nm, the number of real contact particles decreased. This is because the larger particles were more likely to separate the surfaces, and the in-contact particles only left larger ones in the particle distribution. When the total contact pressure was small in Figure 4b, the contact pressure of a single particle then increased, which also describes one reason for the larger contact temperature rise of a particle than when the particle diameter was larger in Figure 4a.

**Figure 5:**
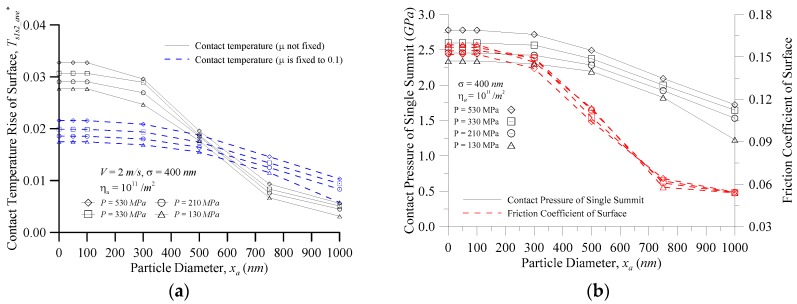
The characteristics of the surface vs. the particle diameter at various contact pressures for (**a**) the contact temperature rise; and (**b**) the contact pressure of a single summit and the coefficient of friction.

Figure 5 shows the characteristics of the surface vs. particle diameter at various contact pressures for (a) the contact temperature rise; and (b) the contact pressure of a single summit and the coefficient of friction when *σ* = 400 nm, *η_a_* = 10^11^/m^2^, and *V* = 2.0 m/s. Figure 5a shows the temperature rise comparison between the coefficient of friction fixed at 0.1 and the non-fixed coefficient of friction. In the case of smaller particles, the change of the real contact temperature rise was greater than that of the contact temperature rise for the general assumption of *μ* = 0.1; and the smaller the particle diameter, the greater the error of the surface temperature rise. When *x_a_* = 0 nm (that is, two-body contact, no third particle), *P* = 530 MPa, T^*^_s__1*s*2*_ave*_ increased from 0.022 to 0.033 (i.e., 36.5 K to 55.3 K) because the unit friction heat flux input is *μPV*, and the smaller particles easily sank into the trough of the surface so that the external load was borne by the surface peak, as illustrated in Figure 5b. The contact pressure of the single summit *P_s_*_1*s*2*,i*_ and the coefficient of friction of the surface *μ_s_*_1*s*2_ were larger, so the contact temperature rise of the surface was higher with the smaller particles, and the contact temperature rise of the surface decreased with an increase in particle size in Figure 5a. In Figure 5b, when the particle diameter was about 650 nm or more, the contact pressure of the single summit *P_s_*_1*s*2*,i*_ and the coefficient of friction of surface *μ_s_*_1*s*2_ increased with increasing contact pressure, so the contact pressure increased, and the contact temperature rise of the surface increased. When the particle diameter was less than 650 nm, the coefficient of friction of the surface did not increase as the contact pressure increased, but each contact pressure of a single summit increased with the increasing contact pressure, so the greater the contact pressure, the greater the contact temperature rise of the surface. As seen in Figure 4 and Figure 5, the surface peak contact pressure was the main impact on the contact temperature rise trends of the surface, and the coefficient of friction of the surface mainly affected the contact temperature rise of the surface. The particle contact pressure was also the main influence on the contact temperature rise trends of the particle, and the coefficient of friction of the particle mainly affected the contact temperature rise of the particle.

**Figure 6:**
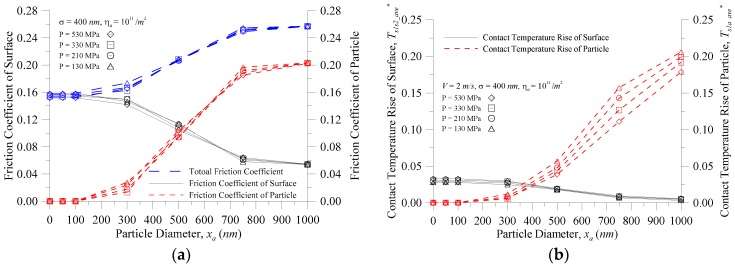
The characteristics of the surface and particle vs. the particle diameter at various contact pressures for (**a**) the coefficient of friction; and (**b**) the contact temperature rise.

Figure 6 shows the characteristics of the surface and particle vs. particle diameter at various contact pressures for (a) the coefficient of friction; and (b) the contact temperature rise when *σ* = 400 nm, *η_a_* = 10^11^/m^2^, and *V* = 2.0 m/s. As shown in Figure 6a, the coefficient of friction of the surface decreased as the particle size increased, and the coefficient of friction of the particle increased as the particle size increased. When *x_a_* < 500 nm, the total coefficient of friction was dominated by the coefficient of friction of the surface, and at *x_a_* > 500 nm, the total coefficient of friction was dominated by the coefficient of friction of the particle. Figure 6b shows that the changing trend of the contact temperature rise of the surface and the contact temperature rise of the particle with the increasing particle size was similar to that of the coefficient of friction. Based on the discussions of Figure 4 and Figure 6, the coefficient of friction was the main impact factor which affected the contact temperature rise in a three-body contact situation. In engineering applications, we try to seek the surface contact with the surface and the particle contact with the surface where the resulting heat balance is assigned to the particles and the surface, which is the ideal operating condition to avoid the interface from too high a contact temperature point. Figure 6b shows that when the particle size was small, the contact temperature rise of the surface was higher; however, when the particle size was large, the contact temperature rise of the particle was too high. Thus, it can be seen from Figure 6b that, under these operating conditions, the contact temperature equilibrium point was approximately *x_a_* = 380 nm where, at this time, the temperature rise parameter was about 0.038 (a temperature rise of about 59.3 K). Therefore, an effective filter in the process of running debris particles or the foreign particles below a size of 400 nm is in the ideal range to avoid surface damage (wear, scuffing) caused by too high a contact temperature rise. Under these conditions, it is better to choose the filter of some component systems to filter out particles with a diameter of 500 nm or more (i.e., the temperature rise does not exceed about 85.8 K).

**Figure 7:**
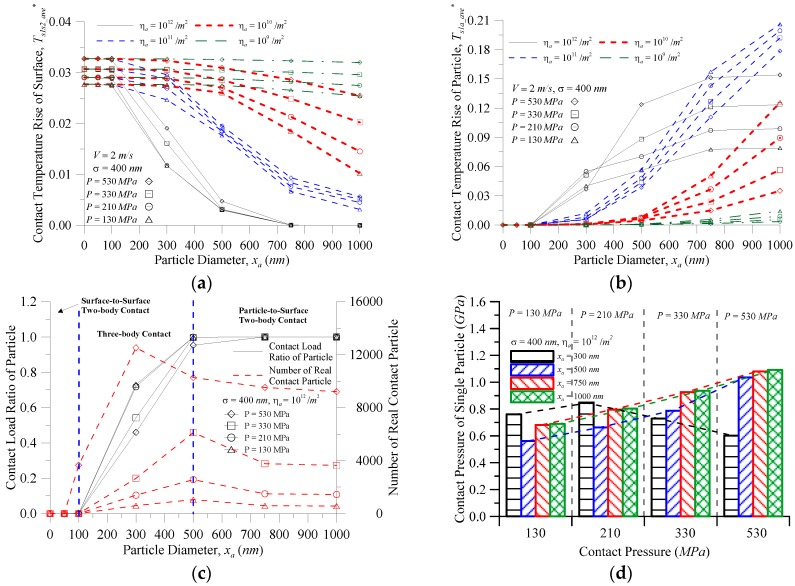
The characteristic of the particle and surface vs. the particle diameter at various contact pressures and area density of particles for (**a**) the contact temperature rise of the surface; (**b**) the contact temperature rise of the particle; (**c**) the contact load ratio of the particle and number of real contact particles; and (**d**) the contact pressure of a single particle.

Figure 7 shows the characteristics of the particle and surface vs. the particle diameter at various contact pressures and the area density of particles for (a) the contact temperature rise of the surface; (b) the contact temperature rise of the particles; and (c) the contact load ratio of the particles and number of real contact particles when *σ* = 400 nm and *V* = 2.0 m/s. Figure 7a shows that the contact temperature rise of the surface increased with increasing contact pressure at any area density of the particles when the particle size was the same. At *x_a_* ≤ 100 nm, at the same contact pressure, with any area density of the particles, the contact temperature rise of the surface was almost overlapping. Under this condition, the particles had barely any effect, and the contact temperature rise of the surface was almost the same as that of the surface under two-body contact. At the same contact pressure, the contact temperature rise of the surface was affected by the particles, and decreased with increases in particle size and the area density of the particles where the larger the particle size or area density of the particles, the greater the decline level. As shown in Figure 7b, when the area density of particles *η_a_* = 10^9^–10^11^/m^2^ with the same particle size, the contact temperature rise of the particles decreased as the contact pressure increased. At the same contact pressure, the contact temperature rise of the particles increased with an increase in the area density of the particles. When the area density of the particles was reduced to 10^9^/**m^2^, the curve showed a steady trend given the sparse distribution of particles on the surface at such a low area density of particles. Most of the external load was still borne by the surface, so the particle size had a limited effect on the external load, and the impact on the temperature rise was also very small. When the area density of particles *η_a_* = 10^12^/m^2^ (at the same contact pressure), the contact temperature rise of the particles increased with an increase in the particle diameter, and rose before stabilizing. However, in the case of the same particle diameter, there was no certain trend of the contact pressure on the contact temperature rise of the particles until the particle diameter increased to 500 nm, then stabilized, and the contact temperature rise of the particles decreased with decreasing contact pressure. When *x_a_* < 300 nm, the temperature rise of the particles showed no certain trend with contact pressure changes as seen in Figure 7c. When the particle size increased from 300 nm to 500 nm, the particles were reduced and the contact load ratio was increased so that the contact pressure increased rapidly for *η_a_* = 10^12^/m^2^ and P = 530 MPa, but the number of real contact particles of other contact pressures still increased so the contact pressure of a single particle was less than *P* = 530 MPa. When *x_a_* > 750 nm, the contact temperature rise of particles *η_a_* = 10^11^/m^2^ was larger than the contact temperature rise of particles *η_a_* = 10^12^/m^2^ (as seen in Figure 4c and Figure 7c) as under the same total load, the area density of the particles increased, as did the number of real contact particles, resulting in a decrease to the contact pressure of a single particle. Therefore, in Figure 7d, under *P* = 130 and 210 MPa, the contact pressure *P* became smaller, whereas the contact pressure of the single particle increased. Figure 7d also shows that the contact pressure of a single particle changed with the contact pressure at the same particle size. This was the same as Figure 7b where the contact temperature rise of the particles changed with the contact pressure. Therefore, the contact pressure of a single particle was the main factor influencing the changing trend in the contact temperature rise of the particles. According to the friction heat flux formula *μPV*, the contact pressure of a single particle is also one of the main factors in producing friction heat between the particles and the surface contact. As indicated in Figure 6, a temperature rise of not more than 85.8 K (with a temperature rise parameter value of 0.055), could be chosen as the filter aperture and also be used to schedule oil changes. Figure 7 depicts that the greater particle size, the greater the area density of the particles, and the contact temperature rise of the particles becomes faster at greater than 0.055. Therefore, the optimum oil change period was when the area density of the particle exceeded 10^12^/m^2^ under this operating condition.

**Figure 8:**
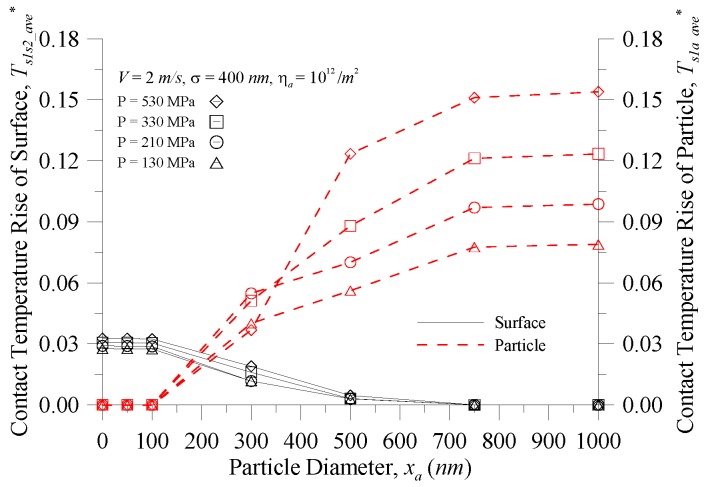
The contact temperature of the surface and particle vs. the particle diameter at various contact pressures.

Figure 8 depicts the contact temperature of the surface and particle vs. particle diameter at various contact pressures for *σ* = 400 nm, *V* = 2 m/s, and *η_a_* = 10^12^/m^2^. As shown in Figure 8, under contact pressure between 130–530 MPa, the equilibrium point between the contact temperature rise of the surface and the contact temperature rise of the particles varied with particle sizes at *x_a_* = 175–225 nm. Therefore, theoretical analysis can predict that under the in-service process of the component, if the particle size can be effectively monitored and controlled, the contact temperature will reach the equilibrium point between the contact interfaces. The contact point will be able to avoid instantaneous excessively higher contact temperatures, resulting in the phenomenon of contact point wear. In addition, when compared with Figure 6, when the area density of particles is increased (10^11^/m^2^ rises to 10^12^/m^2^), the filter is selected to filter out particle diameters of 300 nm or more (i.e., the temperature rise does not exceed ca. 85.8 K as displayed in Figure 6). 

From the results of Figure 4 and Figure 5, any relative movement of the components in a variety of operating conditions will have a different coefficient of friction and different frictional heat generation, thus affecting the micro-contact temperature. In the case of the contact temperature of the surface or contact temperature of the particle, there was a maximum error of about 150% under the non-fixed coefficient of friction and the fixed coefficient of friction, which may result in greater error in more severe operating conditions. Therefore, to make the predicted value of the analysis closer to the actual situation when analyzing the characteristics between the interfaces, the coefficient of friction must be considered in the analysis as the variation of the operating conditions.

The plasticity index is an important indicator of the rough surface contact characteristics, where the greater the value it represents, the higher the chance that plastic deformation will occur at the asperity of the rough surfaces. The plasticity index of the general engineering surface was between 0.7–2.55, and given as:

(19) $$\psi =\frac{E^{*}}{H}\sqrt{\frac{\sigma }{R^{*}}}$$

where E^*^ is the equivalent of Young’s modulus; *H* is the hardness of the soft material; and R^*^ is the equivalent radius of curvature of an asperity.

**Figure 9:**
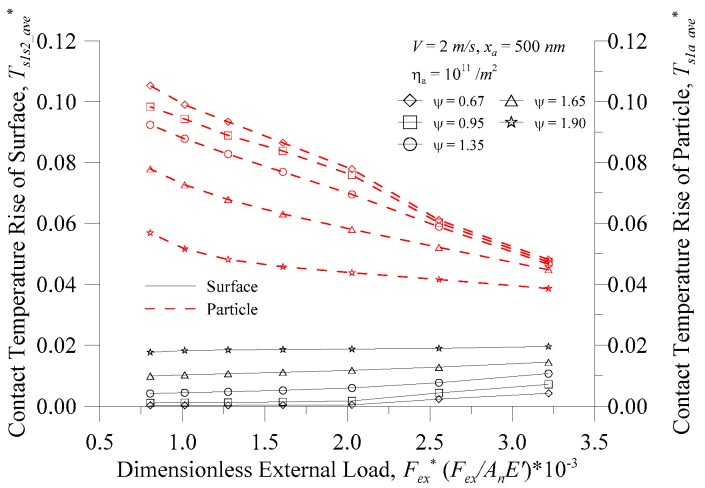
Contact temperature rise of the surface and particle vs. the dimensionless external load at various plasticity indices.

Figure 9 shows the surface and particle temperature rise versus the dimensionless external load (F_ex_^*^) for *V* = 2.0 m/s, *η_a_* = 10^11^/m^2^ and *x_a_* = 500 nm at various plasticity indices (*ψ*). The dimensionless external load and plasticity index are important indicators of micro-contact theory [8]. The dimensionless external load is almost linear with the real contact area [12], and the plasticity index *ψ* is an indicator of the plastic deformation in the real contact area. The greater the value of the plasticity index, the greater the percentage of the plastic deformation area. As indicated in Figure 9, the surface contact temperature increased as the dimensionless external force increased. Under the same external force, the surface contact temperature increased with the increase of the plasticity index. As the plastic index was larger, there was a greater possibility of plastic deformation at the surface peak contact point, and the temperature was higher. While at a low plasticity index value, the external force had little effect on the surface contact temperature, but the surface contact temperature still rose significantly at F_ex_^*^ > 2.0 × 10^−3^. Figure 9 also indicates that the particle contact temperature decreased as the dimensionless external force increased. Under the same external force, the particle contact temperature decreased with the increase of the plasticity index. The plasticity index *ψ* is an important parameter of surface material and rough topography. Particle size and area density of particles are also the main particle properties, and the relationship is discussed below.

**Figure 10:**
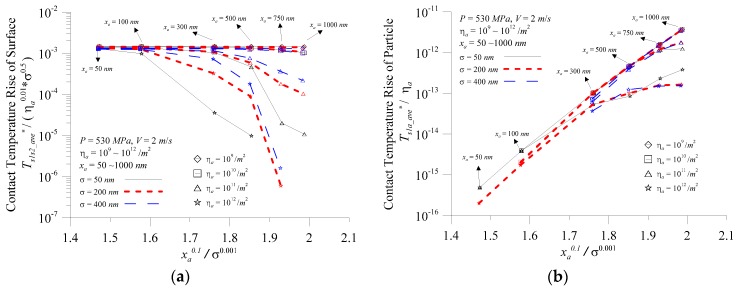
Variation of the contact temperature rise with the ratio of the particle diameter to surface roughness at various surface roughness values and the area density of particles for (**a**) the surface; and (**b**) the particle.

From the previous analysis, when the component is running, the contact pressure, the relative sliding speed, the surface roughness, the generated particle size, the area density of particles, and other operating conditions affect the micro-contact temperature prediction value. Figure 10 depicts the variation of contact temperature rise with the ratio of particle diameter to surface roughness at various surface roughness values and area density of particles for (a) the surface; and (b) the particles when *P* = 530 MPa, *V* = 2.0 m/s, *σ* = 50–400 nm*, η_a_* = 10^9^–10^11^/m^2^, and *x_a_* = 50–1000 nm. Figure 10a indicates *x_a_*^0.1^/*σ*^0.001^ and the predicted value of the surface contact temperature was converted to the surface temperature parameter (*T_s_*_1*s*2_ave_^*^ /*η_a_* ^0.01^*σ^0.5^*) where the surface contact temperature almost overlapped the area density of particles of 10^9^/m^2^. The area density of particles increased to10^10^/m^2^ only in the large *x_a_*^0.1^/*σ*^0.001^, and the surface contact temperature showed a small decline. It showed that the particles affected the surface temperature less significantly and the root mean square roughness was the maximum influence factor of the surface contact temperature. When *η_a_* > 10^10^/m^2^ or more, the surface contact temperature parameter decreased significantly with the increase of the *x_a_*^0.1^/*σ*^0.001^ ratio. When the particle size was the same, the larger the area density of particles, the greater the decrease in surface contact temperature parameters. Figure 10b indicates, except for abnormal *η_a_* = 10^10^/m^2^, the particle contact temperature was almost linear with *x_a_*^0.1^/*σ*^0.001^ when the predicted value of the particle contact temperature was converted to the temperature parameter (*T_s_*_1a_ave_^*^/*η_a_*). As the previous analysis showed, the interface particle temperature was extremely likely to damage the interface performance at *η_a_* = 10^10^/m^2^, but also required general lubrication maintenance to avoid the situation. It also indicated that, under normal circumstances, *x_a_*^0.1^/*σ*^0.001^ was an important influencing factor for particle contact temperature. The particle contact temperature parameter (*T_s_*_1a_ave_^*^/*η_a_*) increased with an increase of the *x_a_*^0.1^/*σ*^0.001^ ratio. However, at high particle concentrations, the rising trend changed slowly, while at a particle concentration of 10^9^/m^2^, there was almost a linear increase.

## 4. Conclusions

This paper considered the existence of abrasive particles in the interface under relative motion. The friction and contact temperature characteristics of different contact loads, particle diameters, and surface roughness were investigated when the moving elements were in contact, and they were compared with the contact temperature where the coefficient of friction was set to a fixed value of 0.1. The following conclusions of the analysis can be made:

1. The average pressure of a single summit and a single particle could be calculated using three-body contact analysis. The main influencing factors of the surface contact temperature were the surface contact coefficient and the average contact pressure of a single summit, and the main influencing factors of the particle contact temperature were the coefficient of friction of the particles and the average contact pressure of a single particle. For contact temperature, the contact pressure of a single summit or particle mainly affected its changing trend, while the coefficient of friction mainly affected its value.
2. Under the operating conditions of this paper, the error between the contact temperature calculated by the fixed coefficient of friction value of 0.1 and the contact temperature calculated by the non-fixed coefficient of friction was up to ca. 150%. Therefore, when any analysis was performed, the coefficient of friction was set to change with the operating conditions to make the analysis closer to the actual situation.
3. Under three-body contact, the surface contact temperature increased with the increase of the *ψ* and the load regardless of the particle size and density; the particle contact temperature increased with increasing particle size.
4. The surface temperature rise was mainly affected by *x_a_*^0.1^/*σ*^0.001^ and *η_a_*^0.01^*σ*^0.5^; the particle temperature rise was mainly affected by *x_a_*^0.1^/*σ*^0.001^ and *η_a_*. The surface contact temperature rise parameter had a low area density of particles (10^10^/m^2^ and below), and the *T_s_*_1*s*2_ave_^*^/*η_a_*^0.01^ *σ*^0.5^ was almost fixed. In addition to the abnormal area density of particles of10^12^/m^2^ or more, the particle contact temperature rise parameter *T_s_*_1a_ave_^*^/*η_a_* was almost linear with *x_a_*^0.1^/*σ*^0.001^.
5. This paper showed that when 0 < *F_s_*_1a_^*^ < 1, the contact interface was a three-body contact. In this condition, the external load part was subjected to the rough crest and the other part was subjected to the particle, and it was possible to prevent frictional heat from locally occurring on the rough crests or particles, resulting in the micro-contact temperature reaching the material failure temperature.

## Figures and Tables

**Table 1:** Mechanical properties.

Property	Value
Hardness of surface 1, *H_s_*_1_ (GPa)	6.3
Hardness of surface 2, *H_s_*_2_ (GPa)	5.8
Young's modulus of surface 1, *E_s_*_1_ (GPa)	210
Young's modulus of surface 2, *E_s_*_2_ (GPa)	197
Young's modulus of particle, *E_a_* (GPa)	197
Poisson ratio of surface 1, *υ_s_*_1_	0.27
Poisson ratio of surface 2, *υ_s_*_2_	0.29
Poisson ratio of particle, *υ_a_*	0.29
Shear modulus, *G* (GPa)	80.0
Thermal conductivity of surface 1, *K_s_*_1_ (W/m·K)	46.6
Thermal conductivity of surface 2, *K_s_*_2_ (W/m·K)	26.6
Thermal conductivity of particle, *K_a_* (W/m·K)	26.6
Specific heat capacity of surface 1, *C_ps_*_1_ (J/kg·K)	475
Specific heat capacity of surface 2, *C_ps_*_2_ (J/kg·K)	460
Specific heat capacity of particle, *C_pa_* (J/kg·K)	460
Density of surface 1, *ρ_s_*_1_ (kg/m^3^)	7850
Density of surface 2, *ρ_s_*_2_ (kg/m^3^)	7800
Density of particle, *ρ_a_* (kg/m^3^)	7800
